# Treatment of dairy cows with PGF2α or NSAID, in combination with antibiotics, in cases of postpartum uterine inflammation

**DOI:** 10.1186/1751-0147-54-45

**Published:** 2012-08-10

**Authors:** Julia Jeremejeva, Toomas Orro, Andres Waldmann, Kalle Kask

**Affiliations:** 1 Department of Therapy, Institute of Veterinary Medicine and Animal Sciences, Estonian University of Life Sciences, Tartu, 51014, Estonia; 2 Department of Animal Health and Environment, Institute of Veterinary Medicine and Animal Sciences, Estonian University of Life Sciences, Tartu, 51014, Estonia; 3 Department of Reproductive Biology, Institute of Veterinary Medicine and Animal Sciences, Estonian University of Life Sciences, Tartu, 51014, Estonia

**Keywords:** Metritis, Treatment, PGF2α, NSAID, Dairy cattle

## Abstract

**Background:**

The aim of the study was to test the effect of two treatments in cases of acute puerperal metritis (APM) and clinical metritis (CM).

**Methods:**

Cows with APM and CM (n = 40)) were matched according to plasma fibrinogen levels (Fb) into three groups. Two negative control groups D (n = 11) and E (n = 17) were composed of healthy cows. The proportion of animals with APM and CM was similar within the groups. Treatment was started on the 3rd day *postpartum* (PP). In group A (n = 15), intramuscular (i.m.) administration of ceftiofur was used for five days in combination with flunixin for three days. Group B (n = 15) received i.m. administration of ceftiofur for five days followed by two injections of prostaglandin F2_α_, with an interval of 8 h, on the 8th day PP. Group C (n = 10) served as a control group with no treatment. The general health status, body temperature (BT) and vaginal discharge were evaluated daily. Endometrial biopsies for bacteriology were taken once a week for seven weeks PP. Blood samples for the analysis of acute phase proteins were collected once a week for six weeks PP. Samples for progesterone analysis were taken twice a week for seven weeks PP. Fertility performance data were recorded.

**Results:**

The area under the curve of BT was higher in group B than in group D cows (P < 0.05). No differences were found for vaginal discharge. There were no differences in bacterial growth, start of ovarian activity or serum amyloid-A or fibrinogen levels among the groups. The haptoglobin concentration was higher in the first and second weeks PP in group B compared with the other groups (P < 0.05). The number of days open was higher in group A than in both groups B and D (P < 0.05). The pregnancy rate after the first two services was higher (P < 0.05) in groups B and D than in groups A and C. The number of services per pregnancy was lower in group B than in group C (P < 0.05).

**Conclusions:**

Regardless of more severe uterine inflammation found in animals from group B, these cows showed the same fertility parameters as healthy animals.

## Background

Acute puerperal metritis (APM) and clinical metritis (CM) occur in the early postpartum (PP) period. They constitute a widespread problem in dairy farming that causes high economic losses [1]. Therefore, treatment of PP uterine inflammation is one of the most frequently investigated topics in dairy cow health. Regardless of the large number of scientific publications devoted to the treatment of APM and CM, the question of the most optimal treatment scheme is still open.

During normal uterine involution process after parturition a variety of aerobic and anaerobic bacteria, growing in the uterine lumen, are removed through a range of uterine defence mechanisms [2]. These mechanisms are effective in combination with good preventive measures, such as ensuring cows’ adequate immune status and the correct energy balance around calving, together with good hygiene. Otherwise, following contamination of the uterine lumen by such pathogens as *Arcanobacterium Pyogenes, Fusobacterium necrophorum, Prevotella species and Escherichia coli* can cause inflammation, which can involve the uterine mucosa alone, or also inflammation of the uterine wall [2,3]. In some cases is very difficult to differentiate APM from CM. Results from previous studies [3,4] showed that in some cases no pyrexia was found in cases of APM with severe bacterial infection. So, considering that APM and CM have the same etiology, and the difficulty in differentiation between these diseases, they could both be treated using the same treatment scheme.

So, diagnosis of APM or CM means that inflammation of uterine wall is caused by pathogens. Treatment of these uterine inflammations should include using of intrauterine or parenteral antibiotics. However, the use of intrauterine antimicrobial therapy does not reduce the signs of systemic illness caused by APM; although intrauterine administration produces higher drug concentrations in the endometrium, there is little penetration into the deeper layers of the uterus or the other reproductive tissues [5,6]. Additionally, the use of intrauterine therapy in combination with parenteral antibiotics has shown no difference when compared with the use of parenteral administration alone [7]. Drillich et al. reported that the use of intramuscular (i.m.) injections alone can be less expensive for farmers [8]. Therefore, the use of parenteral antibiotics for the treatment of APM and CM may be more sensible in practice than intrauterine administration.

Nonsteroidal anti-inflammatory drugs (NSAID) and prostaglandin (PG) F2_α_ are often used in addition to antibiotics. However, the results of studies in which the effects of these drugs have been tested are not consistent [9,10]. The results of studies concerning the use of PGF2_α_ alone show the same problem. Some investigations showed a clear positive effect of PGF2_α_ on fertility parameters [11-13], while others found no effect of PGF2α on fertility parameters [14-17].

The objective of this study was to compare systemic antibiotic treatment in combination with either flunixinmeglumine (an NSAID) or PGF2_α_ for the treatment of APM and CM. The effect of these combinations on clinical and inflammatory parameters and fertility traits on a commercial dairy herd were investigated.

## Materials and methods

Study was performed according to approval of an ethics committee of animal experiments of Estonian Ministery of Agriculture from 04.09.2007 ref no. 7.2-11/6940 - 1.

### Farm and animals

The study was conducted from September 2007 to February 2008 on a loose-housing commercial dairy farm with 600 cows. All cows were fed the same diet in the form of a total mixed ration (TMR). The TMR consisted of a mixture of grass silage and concentrate. The cows were milked three times a day using a 2 × 20 parallel milking parlour.

Multiparous late pregnant Estonian Holstein Friesian cows (n = 68) with mean milk production during the previous lactation of 10,233 kg energy-corrected milk, and which were expected to calve during the following two months, were used for the study.

### Diagnosis and treatment

Animals with clinical mastitis, arthritis, hoof problems and other clinical diseases other than APM and CM were excluded from the study. The diagnosis of APM and CM was made on the 3rd day PP using vaginal and rectal examination and body temperature measurements. Clinical metritis was defined if an atonic uterus with purulent or mucopurulent vaginal discharge was found, without changes in general health condition. A diagnosis of APM was confirmed when increased body temperature (≥ 39.5°C), anorexia, an enlarged and atonic uterus and odiferous vaginal discharge with the presence of pus were found [2,18]. To ensure minimal variation of the initial response to inflammation of the uterus among the treatment groups, stratified randomisation, on the basis of the plasma fibrinogen (Fb) measurements on day 3 PP, was used. The cows were divided into subgroups with minimal variation in fibrinogen concentration (for example, a subgroup with concentrations between 5.1 g/l and 6.0 g/l was used). Animals in each subgroup were assigned randomly to the three experimental groups. Treatment of the animals was started on the 3rd day PP. Group A (n = 15) was treated by s.c. injection of 1 mg/kg Ceftiofur (Exenell RTU®, Pharmacia Animal Health) for five days, in combination with simultaneous injections of 1.1 mg/kg Flunixin (Finadyne® vet., Schering-Plough Animal Health) for the first three days. Animals in group B (n = 15) were treated by injection of 1 mg/kg Ceftiofur (Exenell RTU®, Pharmacia Animal Health) for five days followed by two injections of 25 mg PGF_2α_ (Dinoprost; Dinolytic®, Pfizer Animal Health), with an interval of 8 h, on the 8th day PP (treatment scheme described by Melendez et al. [13]). Group C (n = 10) served as a positive control group with no treatment.

Animals with a body temperature of < 39.5°C, tonic uterus, clear or some blood containing (normal lochia) vaginal discharge without any pus, and absence of anorexia, were diagnosed as healthy cows and incorporated negative control group D (n = 11). An additional negative control group, E (n = 17), was formed to allow examination of the normal Fb level in healthy cows.

### Clinical examination

The cows were observed daily for morning body temperature (BT) and health problems. Body temperature measurements started on the day of parturition, but statistical analysis was performed from day 3 PP. Temperature measurements were made during the first two weeks PP. Health problems were recorded starting from two weeks before parturition until the end of the experimental period at seven weeks PP. Evaluation of the presence and character of vaginal discharge on the vulva, perineum, or tail was assessed daily for each animal for seven weeks PP. The appearance of vaginal discharge was scored as according to Bekana et al. [19] as follows: 0 = no discharge; 1 = clear mucus; 2 = mucus with the presence of pus; 3 = viscous purulent material; 4 = viscous haemopurulent discharge; 5 = watery mucohaemorrhagic malodorous secretion.

### Bacteriological sampling and examination of the uterus

Biopsies from the uterine endometrium were collected from animals with CM and APM (groups A, B and C) once a week over a period of seven weeks. If two sequential samples from the same animal were determined to be bacteriologically negative, assessed as a uterus clear of bacteria, collection of biopsies from this animal stopped. The first biopsy was taken between days 3 and 7 PP. Samples were collected according to the methods described by Bekana et al. [19] and Kask et al. [20]. At the start of sampling the cow`s tail was secured, faeces were removed from the rectum, and the perineal region and vulva were thoroughly washed and disinfected. The vulva lips were parted to introduce an externally sterile protective stainless steel tube. The instrument was advanced into the vagina and fixed in the external opening of the cervix. A sterile long guarded culture instrument was advanced into the uterus by cervical manipulation, and opened. A milled cavity with a sharpened edge, forming a curette about 2 cm in length, 0.5 cm from the rounded tip, was located with the forefinger of the hand in the rectum and biopsy was obtained from the endometrium. The biopsies were placed immediately in 10 ml of a freshly prepared thioglycollate medium (LAB M, Bury, England) for transportation to the laboratory. Aliquots of 0.01 ml of each sample (in the thioglycollate medium) were spread on Columbia blood agar plates containing 5% bovine blood. The plates were cultivated aerobically and anaerobically and examined after 24 and 72 h respectively. Identifications were made according to Bergey’s Manual of Systematic Bacteriology [21]. To determine the species of the isolates, BBL Crystal^TM^ (Becton, Dickinson and Company, Maryland, USA) miniaturized biochemical test systems (Gram positive, Enteric/nonfermenters and Anaerobe ID kits (BD BBL Cristal^TM^ Identification Systems)) were used. The intensity of bacterial growth on the plates was interpreted as follows: 0 = no bacteria isolated, 1 = mild growth (1–20 isolated colonies per 10 μl of cultivated material), 2 = moderate growth (21–40 isolated colonies per 10 μl of cultivated material), 3 = heavy growth (≥41 isolated colonies per 10 μl of cultivated material).

### Blood samples

Blood samples for analysis of acute phase proteins (APP) (serum amyloid-A (SAA) and haptoglobin (Hp)) were taken from coccygeal vessels using heparinized venoject glass tubes (Terumo Europe N. V. Leuven, Belgium). Collection was started nine days before parturition and was performed once before parturition, and once a week for six weeks after parturition. After immediate centrifugation, about 5 ml of plasma was removed and stored at −18°C until analysis. Whole blood for Fb analyses was taken once a week during the entire experimental period, starting on the 3rd day PP.

For the determination of progesterone (P_4_), plasma samples were collected twice a week. Collection was started from day 10 PP and continued until day 55 PP. Plasma was stored at −18°C until analysis.

### Methods of analysis of APP

The fibrinogen concentration in plasma was measured by the heat precipitation method [22]. Microhaematocrit tubes were filled with EDTA whole blood and sealed at one end. After centrifugation for 3 min at 6.000 r/min, the tubes were placed in a water-bath at 56.3°C for 3 min. The plasma Fb concentration (g/l) was measured after a second centrifugation for 3 min, by calculating the percentage of the precipitated Fb column relative to the length of the plasma column. Plasma Hp was determined using the haemoglobin binding assay described by Makimura and Suzuki [23], with the modification of tetramethylbenzidine (0.06 mg/ml) used as a chromogen [24]. Pooled and lyophilized aliquots of bovine acute phase serum were used to create standard curves. To calibrate the assay, a bovine plasma sample with a known Hp concentration provided by the European Commission Concerted Action Project (number QLK5-CT-1999-0153) was used. The range of the standard curve was 0.04–1.16 g/l. If a sample’s Hp concentration was higher, the sample was diluted with isotonic saline and re-assayed. The intra- and inter-assay coefficients of variation were <12% and <11% respectively. Concentrations of serum amyloid A (SAA) in plasma were measured with a commercially available ELISA kit (Phase SAA kit, Tridelta Development Ltd.) according to the manufacturer’s instructions. The detection limit of the assay for bovine samples is 0.3 mg/l. The intra- and inter-assay coefficients of variation were <7% and <14% respectively.

### P_4_ analysis

Plasma P_4_ was determined with a commercially available ELISA kit (EIA-1561, DRG Instruments GmbH, Germany) according to the manufacturer’s instructions. The intra-assay precision at P4 concentrations of 0.28 and 5.2 ng/ml were 6.76 and 7.76%, respectively. The inter-assay precision at P4 concentrations of 0.28 and 4.9 ng/ml were 7.28 and 2.78%, respectively.

The plasma P_4_ concentration was used to determine the number of days to the first luteal response PP, defined as the first two consecutive measurements of P_4_ concentrations > 1 ng/ml.

### Fertility analysis

For evaluation of fertility performance, the interval from calving to the first insemination (days to first service, DFS), first service conception rate (FSCR,%), two service (first and second) conception rate (TSCR,%), interval from calving to successful insemination (days open, DO) and number of services per pregnancy (NSP) were recorded for all cows.

### Statistical analyses

Linear random-intercept models were used to explore differences in APP concentrations between the treatment groups. Treatment group, sampling time (week), their interaction and diagnosis (APM or CM) were included as fixed factors in the initial models. Diagnosis was not included in the final models because it was non-significant. The cow was included as a random factor. Isotropic spatial exponential correlation structures were used to model serial correlations of repeated measurements within cows. Logarithmic transformation of SAA and inverse-root transformation for Hp were used. Differences in bacterial counts and vaginal discharge among groups were tested using generalized linear mixed models in which a Poisson distribution was used for the response variables. The cow was included as a random factor. Linear time (weeks) as a continuous variable, and time interactions with treatment group and diagnosis (APM or CM), were included as fixed factors. Overall time trend differences between challenges were tested with an F-test. Isotropic spatial exponential correlation structures were used for modelling serial correlations of repeated measurements within cows in both mixed models. The nlme-package [25] with statistical software R 2.5.0 [26] was used for fitting linear random-intercept models, and generalized linear mixed models were fitted using the GLIMMIX procedure [27] software with the SAS/STAT 9.1 (SAS Institute Inc., Cary, NC, USA).

For comparing differences in rectal temperature, the area under the curve (AUC) was calculated for each cow. Analysis of variance, followed by the Bonfferoni correction, was used to compare mean AUC values among the treatment groups. Logistic regression was used to evaluate differences among treatment groups in days to the first luteal response (before 50 days PP or after 50 days PP), first service conception (pregnant or not) and second service conception results. Poisson regression was used to evaluate group differences in NSP, and linear regression was used to evaluate group differences in DO after logarithmic transformation of DO. These analyses were made using STATA 10.0 (Stata Corporation, Texas, USA) software. Data are presented as means ± SEM.

Power analyses of treatment effect to fertility parameters were made retrospectively.

## Results

One animal from group B was culled from the herd before the end of the sampling period because of polyarthritis and was excluded from the study. There was a small variation in the proportion of animals with diagnosed APM within groups (6 of 15 (40%), 5 of 14 (35.7%) and 4 of 10 (40%) in groups A, B and C, respectively). The other animals from groups A, B and C showed signs of CM.

### Clinical parameters

**Figure 1:**
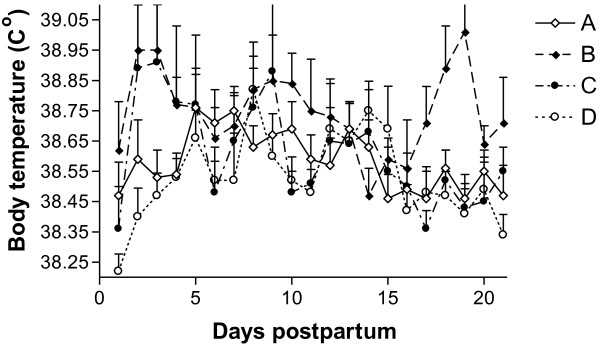
**Mean (±SEM) daily body temperature of cows treated with a combination of parenteral antibiotic and NSAID (group A) and with a combination of parenteral administration of antibiotic and PGF2**_**α**_**(group B), in comparison with the non-treated control group (group C) and healthy cows (group D).**

The raw data of daily body temperature is presented in Figure 1. The area under the mean BT curve (AUC) was highest in group B (AUC = 775.49 ± 1.22). It was significantly higher than in groups D (AUC = 770.85 ± 0.85, P = 0.04) and showed the trend to be higher than in group A (AUC = 771.41 ± 0.96, P = 0.056). The area under the BT curve in group C was similar to that in group A (AUC = 771.31 ± 1.74), but no differences were found in comparison with the other groups.

**Figure 2:**
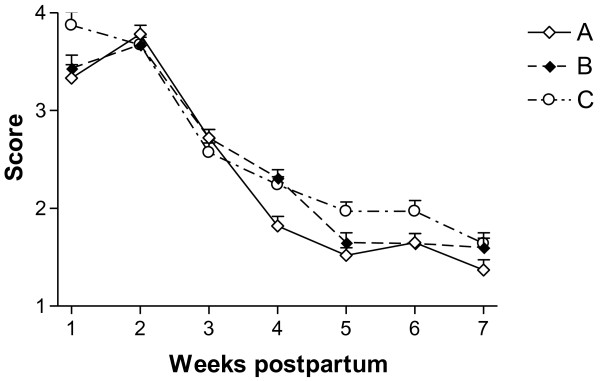
**Mean (±SEM) score for vaginal discharge of cows treated with a combination of parenteral antibiotic and NSAID (group A) and with a combination of parenteral administration of antibiotic and PGF2**_**α**_**(group B), in comparison with the non-treated control group (group C) and healthy cows (group D).** Vaginal discharge was scored as follows: 5 = watery mucohaemorrhagic malodorous secretion; 4 = viscous haemopurulent discharge; 3 = viscous purulent material; 2 = mucus with the presence of pus; 1 = clear mucus; 0 = no discharge.

The mean score for vaginal discharge in animals with a diagnosis of APM at the beginning of the study was higher than in animals with CM (P < 0.001). The decrease in the vaginal discharge score throughout the study period did not differ between cows with APM and CM (P = 0.23). No differences among the experimental groups in vaginal discharge were found (Figure 2).

### Bacteriological examination

**Figure 3:**
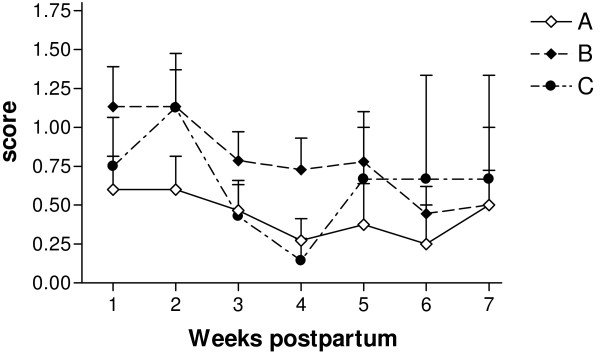
**Mean (±SEM) score of bacterial growth in cows treated with a combination of parenteral antibiotic and NSAID (group A) and with a combination of parenteral administration of antibiotic and PGF2**_**α**_**(group B), in comparison with the non-treated control group (group C) and healthy cows.** The number of bacterial colonies was scored as follows: 0 = no bacteria isolated, 1 = mild growth (1–20 isolated colonies), 2 = moderate growth (21–40 isolated colonies), 3 = heavy growth (≥41 isolated colonies).

The results for bacteriological growth are presented in Figure 3. A total of 201 biopsy specimens were collected, of which 101 were positive for bacteria; the remaining 100 biopsy samples were found to be bacteriologically negative. The highest number of positive samples was found in group B (49 of the total of 101 positive samples). Nine consistently negative animals out of the 40 in groups A, B and C were found. From the 101 positive samples, 15 showed mixed infections: two samples from group A, 10 from group B, and three samples from group C. The most frequent isolates in the positive samples were *Bacteroides* spp. (30.7%), *Corynebacterium* spp (21.8%). and *A. pyogenes* (10%). The number of positive samples and the intensity of bacterial growth decreased with every week PP (P < 0.001 and P < 0.001, respectively). Animals with an APM diagnosis showed more intensive bacterial growth in all groups (P = 0.04). However, the number of positive samples, intensity of bacterial growth and their time trends did not differ between groups.

### Acute phase proteins

**Figure 4:**
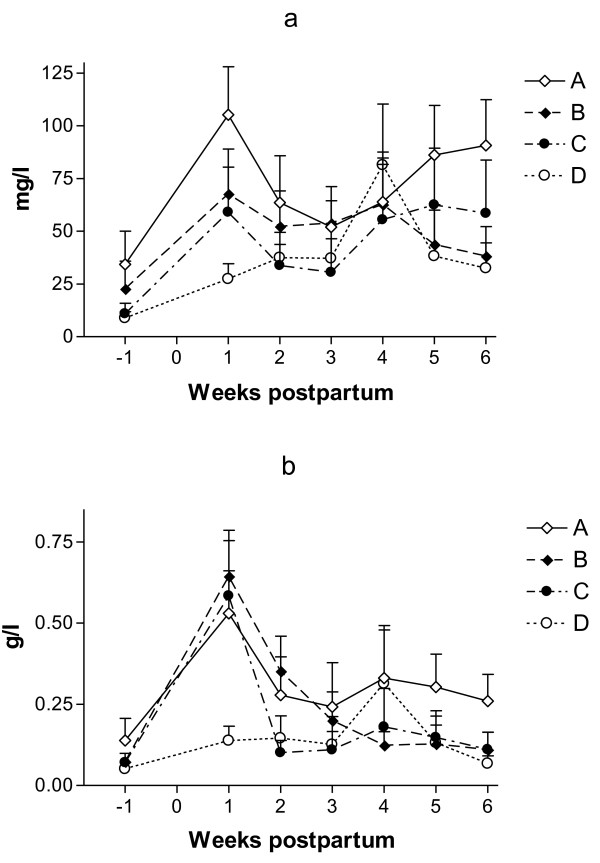
**Mean (±SEM) concentrations of serum amyloid A (a) and haptoglobin (b) in cows treated with a combination of parenteral antibiotic and NSAIDs (group A) and with a combination of parenteral administration of antibiotic and PGF2**_**α**_**(group B), in comparison with the non-treated control group (group C) and healthy cows (group D).**

The maximal levels of SAA were seen in all groups during the 1st week after parturition (P < 0.001 in all groups; Figure 4a). The differences in levels among groups, considering their basal levels, were not significant.

The maximal levels of Hp were also seen in all groups during the 1st week after parturition (Figure 4b). Hp levels in group B in the 1st week PP were higher than in groups A and D (P = 0.047 and 0.032, respectively). The levels in group B also remained higher in the 2nd week, when compared with groups A and C (P = 0.013 and 0.011, respectively).

**Figure 5:**
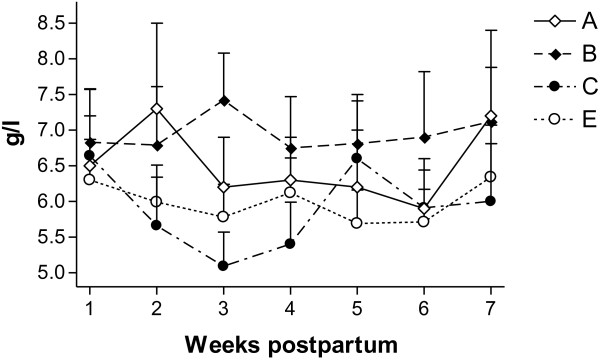
**Mean (±SEM) concentrations of fibrinogen in cows treated with a combination of parenteral antibiotic and NSAIDs (group A) and with a combination of parenteral administration of antibiotic and PGF2**_**α**_**(group B), in comparison with the non-treated control group (group C) and healthy cows (group E).**

Figure 5 shows the change in the group means for Fb levels. There was no difference between the groups in the change of levels from the first Fb values.

There was no difference in the levels of APP between animals with APM and those with CM.

### Progesterone

One animal out of 51 was culled from the herd because of polyarthritis before the end of the sampling period and was excluded from the analysis of the start of ovarian activity. More than half of the animals (29 out of 50) showed the start of ovarian activity (first luteal phase postpartum) during the first 50 days PP. The majority of animals in groups C and D showed an increase in progesterone of above 1 ng/ml: 70% (7 of 10) and 63% (7 of 11), respectively. Luteal activity was seen in fewer animals from groups A and B during the first 50 days PP: 53% (8 of 15) and 50% (7 of 14), respectively. No statistical difference between the groups was found with regard to the start of ovarian activity.

### Fertility parameters

Of the total of 51 cows, fertility data were obtained from 39. The data for 12 animals (two cows in group A, seven in group B, two in group C, and one in group D) were absent because the cows had been culled owing to health problems not associated with reproduction (leg problems, pneumonia and other diseases) during the period between 5 weeks and 6 months PP.

Table 1 provides a summary of the parameters of reproductive performance. The animals in group A showed a longer period of DO than those in groups B and D (P = 0.016 and 0.039, respectively). There was no difference among the groups in the number of days to first service and the first service conception rate. The two-service conception rate was significantly higher in group D than in both groups A and C (P = 0.049 and 0.035, respectively). It was not possible to perform analysis of the TSCR in group B using logistic regression because of a 100% pregnancy rate. The number of services per pregnancy was detected as minimal in group B in comparison with group C (P = 0.027). It was also smaller in group B than in groups A and D, this difference was close to significance (P = 0.054 and P = 0.075, respectively). Animals diagnosed with APM showed the same DFS, DO, FSCR, TSCR, and NCP as animals with CM.

**Table 1:** **Fertility parameters of cows treated with a combination of parenteral antibiotic and NSAID (group A) and with a combination of parenteral administration of antibiotic and PGF2**_**α**_**(group B) in comparison with the non-treated control group (group C) and healthy cows (group D)**

**Experimental group**	**Days to first service**	**Number of days open**	**First service conception rate (%; number of pregnant animals)**	**Two services conception rate (%; number of pregnant animals)**	**Number of services per pregnancy**
A (n = 13)	82.3 ± 4.0 ^a^	170.4 ± 20.8 ^a^	23; 3/13 ^a^	46; 6/13 ^a^	2.85 ± 0.42 ^ab^
B (n = 8)	98.6 ± 13.6 ^a^	120.6 ± 21.9 ^b^	50; 4/8 ^a^	100; 8/8	1.5 ± 0.19 ^a^
C (n = 8)	76.6 ± 5.0 ^a^	157.0 ± 20.3 ^ab^	37; 3/8 ^a^	37; 3/8 ^a^	3.25 ± 0.88 ^b^
D (n = 10)	85.8 ± 5.9 ^a^	115.3 ± 15.3 ^b^	30; 3/10 ^a^	90; 9/10 ^b^	1.9 ± 0.28 ^ab^

^a,b^ Groups with different superscripts within the column differ significantly (P < 0.05).

The results of retrospective power analyses based on the observed differences of FSCR, TSCR and DO between treatment groups showed that the power of the experiment was 40-50%. To achieve 80% power the size of each group should be at least 22 animals.

## Discussion

The retrospective analysis of optimal sample size and power analysis showed that sample size of our study is not enough for good power of the study. The treatment studies are usually expensive and work and time requested. Granted of afore mentioned reasons, we unfortunately, could not involve more animals. However, we got a statistically significant difference between treatment groups with P-value less than 0.05. Differences between groups were so considerable, that significant differences were found even with the small group sizes used in this experiment. The probability of Type I error (getting of false positive result) is several times less, than probability of Type II error (getting of false negative result) [28], and the possibility of this error was minimized as statistically significant P-values were achieved. So, regardless of small sample size used in the study, the results of the study can be regarded as reliable.

The first purpose in the treatment of APM and CM is to support the cow’s wellbeing and reduce the period of depression and loss of appetite. Reducing the clinical signs of disease (normalization of BT and vaginal discharge), followed by a decrease in the systemic inflammatory reaction, and elimination of bacteria from the uterine lumen, are the physiological indicators of the disappearance of these diseases. Non-steroidal anti-inflammatory drugs, due to their antipyrectic and anti-inflammatory effects, should reduce the clinical signs and inflammatory parameters that accompany APM and CM, as well as signs of intoxication. It is especially important in the case of severe cases of APM and toxification due to lipopolysaccharides from bacteria (*E.coli* and others). These conditions may be fatal if not treated. The use of NSAIDs in the present study, however, did not help to decrease the BT more rapidly than occurred in non-treated animals. The process of normalisation of vaginal discharge and decrease of APP levels in the group treated with flunixin was not optimal, but it also was not worse than in the non-treated group. The number of bacteriologically positive samples from uterine biopsy, intensity of bacterial growth and their time trends, and the start of ovarian activity did not differ among the groups. These results are in agreement with a study by Drillich et al. [10], who evaluated the effects of a single administration of flunixinmeglumine in addition to systemic antibiotic treatment in cows with acute puerperal metritis and did not detect any effect of flunixin on clinical cure.

The same study [10] showed the absence of an effect of flunixin on reproductive performance compared to another treated group. In the study of Amiridis et al. [9], animals with puerperal metritis treated by flunixin meglumine, in addition to systematic antimicrobial treatment, showed more rapid uterine involution and start of ovarian activity than controls, which is not in agreement with the present study. In the current study animals treated by flunixin showed the same interval from calving to the first insemination as other animals. The start of ovarian activity also did not differ between groups. The number of DO in our study was significantly higher in treatment group A in comparison with treatment group B and healthy animals. The pregnancy rate after the first two services, and the number of services per pregnancy in group A, also did not differ from those of the positive control group C. The absence of a positive effect of flunixin administrated in the early PP on fertility could be explained by no effect of flunixin on the ovaries and a weak effect on the uterus, in contrast with exogenous PGF_2α_. administrated after a decrease in endogenic PG levels. It is known that as long as the PGF_2α_ release dominates, as occurs during PP uterine inflammation, ovulation does not occur. When the PG metabolite levels have returned to baseline, ovulation can occur [29]. In the study of Königsson et al. [30], the early (3–6 days PP) flunixin treatment, when the release of PG is high, also suppressed levels of PG metabolites during the treatment period of cows with PP uterine inflammation. But this suppression was not complete, and not so severe, as in case of administration of flunixin at the end of the second week PP. Lindell et al. [31] and Madej et al. [32], however, showed a significant negative correlation between the duration of PG release and uterine involution. Guiltbault et al. [33], in contrast, did not detect a negative effect of flunixin meglumine on uterine involution. In the present study, the number of DFS was the same in all groups, which indicated simultaneous uterine involution in all groups. Therefore: healthy, diseased and treated, and diseased and non-treated animals needed the same time to first insemination. These results are in agreement with studies by Gautam et al. [34] and Barlund at al. [35], in which animals with clinical uterine inflammation did not need more time to first insemination than healthy cows. The start of ovarian activity in the present study was also the same in all experimental groups.

It is interesting that animals in non-treated group C did not show a larger number of DO than healthy animals. Dolezel et al. [36] reported that the probability of conceiving from the first insemination was the same in animals with AMP and animals with clinically normal vaginal discharge. Similar results were obtained in the current study, where the FSCR was the same in all groups. Therefore, the probability of becoming pregnant after the first insemination was the same in the group treated by flunixin and the group treated by PGF2_α_, as well as in healthy and untreated animals. Hendricks et al. [16] also showed that treatment using PGF2_α_ did not alter the probability of pregnancy at first insemination.

However, the TSCR in the present study was highest in the group treated by PGF_2α_ in addition to parenteral antibiotics, and in the healthy control group. Animals from group B showed the same positive result for NSP as control group D. These results are in agreement with some previous studies in which the mean number of services per pregnancy was lower in healthy animals [34,35], which means that cows from group B in the present study probably recovered from uterine inflammation. It is known that prostaglandins enhance uterine defence mechanisms, help to promote uterine contractility, contribute to uterine involution and help to shorten periods of bacterial infection [11-13]. Hirsbrunner et al. [15] in their study, however, did not detect any significant effect on the fertility parameters measured (DFS, DO, NSP) using PGF2_α_ in dairy cows in the PP period. In addition, Mejia and Lacau-Mengido [17], who used a PGF2_α_ analogue for treatment of endometritis, detected a negative effect of PGF2_α_ on the DFS and pregnancy rate by day 90 PP in multiparous cows.

The only negative effect of PGF2_α_ in the present study was found for inflammatory parameters and the clinical conditions of the animals (the highest BT and Hp concentration in the early PP period). Use of PGF2_α,_ in combination with systemic antibiotic for the treatment of APM, in a study by Jeremejeva et al. [37], also did not improve the condition of the uterus during the late PP period. Bonnet et al. [12], in contrast, reported that treating cows with PGF2_α_ reduced the incidence of vaginal discharge, decreased the diameter of the uterine horns, reduced inflammation and fibrosis in the endometrium and minimized the possibility of isolation of *Actinomyces pyogenes* from endometrial biopsy samples taken on the 40th day PP. In the present study, vaginal discharge, intensity of bacterial growth and the start of ovarian activity in animals treated by PGF2_α_ were not better than in the other groups.

Choosing a treatment strategy for APM and CM must be based on the animal welfare and economic aspects. It is important not to over-diagnose PP uterine inflammations and is possible that in the case of less severe cases of CM PGF2_α_ alone should be used.

## Conclusions

Treatment of APM and CM using NSAID in addition to parenteral antibiotic did not improve clinical cure, inflammatory parameters or elimination of bacteria from the uterus. Animals from the group given NSAID showed the same fertility parameters (length of interval from calving to first insemination, first service conception rate, conception rate after two services, interval from calving to successful insemination and number of services per pregnancy) as non-treated animals. Regardless of more severe inflammation that was detected in animals from the group treated by parenteral administration of antibiotic with PGF2_α_ (the highest body temperature and concentration of Hp), they showed the same fertility parameters as healthy animals.

## Competing interests

The authors declare that they have no competing interests.

## Authors’ contributions

JJ carried out the study, compiled the results and drafted the manuscript. TO participated in designing the study, statistical analyses of the data and APP analysis. AW performed analysis of P_4_ and KK coordinated the study. All authors were significantly involved in designing the study, interpreting data and composing the manuscript. All authors read and approved the final manuscript.
